# APENDIC-RADS: an ultrasound reporting system for the diagnosis of acute appendicitis

**DOI:** 10.31744/einstein_journal/2024AO1164

**Published:** 2024-11-26

**Authors:** Marcos Roberto Gomes de Queiroz, Victor Arantes Jabour, José Leão de Souza, Milena Ribeiro Paixão, Paulo Savoia Dias da Silva, Davi Wen Wei Kang, Gaby Cecilia Yupanqui Guerra Barboza, Guilherme Muniz Bourroul, Juliana Maria Haddad de Lamare, Irline Cordeiro de Macedo Pontes, Gabriela Cauper de Carvalho Pereira, Wanessa Rolando Roselli, Marcelo Rocha Corrêa da Silva, Antonio Rahal, Cesar Augusto Passos Braga, Miguel José Francisco

**Affiliations:** 1 Hospital Israelita Albert Einstein São Paulo SP Brazil Hospital Israelita Albert Einstein, São Paulo, SP, Brazil.; 2 Universidade de São Paulo Faculdade de Medicina Hospital das Clínicas São Paulo SP Brazil Hospital das Clínicas, Faculdade de Medicina, Universidade de São Paulo, São Paulo, SP, Brazil.

**Keywords:** Appendicitis, Ultrasonography, Emergency medicine, Interdisciplinary communication

## Abstract

**Objective:**

Abdominal ultrasonography is widely used to evaluate suspected cases of appendicitis. Objective descriptions of the direct and indirect signs of appendicitis result in varied assessments of its likelihood. This study introduces the Appendix Imaging Reporting and Data System (APENDIC-RADS) to standardize the reporting of appendix ultrasound findings.

**Methods:**

This single-center retrospective study included consecutive patients of all ages who underwent abdominal ultrasonography for the investigation of acute appendicitis. The primary outcome was histopathological confirmation of acute appendicitis post-surgery. The imaging findings were classified into five categories: APENDIC-RADS 0, where the appendix cannot be visualized; APENDIC-RADS 1, indicating a normal appendix; APENDIC-RADS 2, describing an appendix that is likely normal but only partially visualized; APENDIC-RADS 3, appendicitis cannot be ruled out due to uncertain features and APENDIC-RADS 4, acute appendicitis.

**Results:**

A total of 747 patients were assessed for suspected acute appendicitis using ultrasonography. Of the diagnosed patients, 52% were male, primarily exhibiting symptoms such as nausea and/or vomiting (60%), right iliac fossa pain (54%), and sudden decompression in the right iliac fossa (24%). Stratification into APENDIC-RADS categories revealed a significant variation in the incidence of acute appendicitis, with incidence rates of 4.5% for category 0 and 0.7%, 2.2%, 11.5%, and 93.5% for categories 1 to 4, respectively (p<0.001). The APENDIC-RADS showed excellent discriminative ability, evidenced by an area under the receiver operating characteristic curve of 0.950 (95%CI=0.899-1).

**Conclusion:**

APENDIC-RADS categorization demonstrated excellent performance in standardizing the ultrasound-determined probability of acute appendicitis. Its implementation could improve physician communication and standardization of patient management.

## In Brief

Acute appendicitis is a common and critical surgical emergency. Abdominal ultrasonography is widely used to evaluate suspected cases of appendicitis. This study introduces the Appendix Imaging Reporting and Data System (APENDIC-RADS) to standardize ultrasound reporting of the appendix. Its implementation could improve physician communication and standardization of patient management.

## Highlights

■ Imaging plays a fundamental role in the diagnosis of acute appendicitis.
■ The study highlights ultrasound's effectiveness in diagnosing appendicitis.
■ APPENDIC-RADS efficiently standardizes the ultrasound evaluation of appendicitis.
■ Using APPENDIC-RADS may enhance communication and optimize medical practices.

## Graphical abstract

**Figure:**
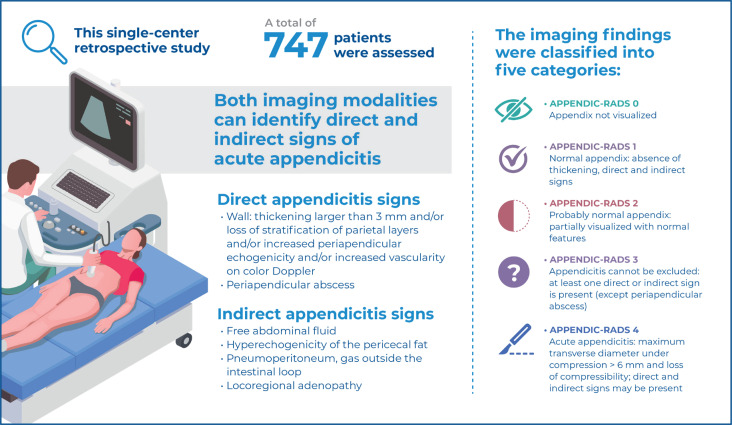

## INTRODUCTION

Acute appendicitis is a common and critical surgical emergency, affecting an estimated 100-200 individuals per 100,000 people annually.^(1,2)^ This condition requires timely and accurate diagnosis to prevent complications such as perforation and abscess formation, which are common in the progression of an untreated appendiceal inflammatory process.^(3–6)^

Ultrasonography and computed tomography (CT) play a pivotal role in evaluating abdominal pain in emergency settings. Computed tomography scans are known for their higher sensitivity and specificity.^(3–5,7)^ However, ultrasonography is favored as the first tool for many patients due to its good accuracy, wide availability, and absence of ionizing radiation.^(8–12)^ Both imaging modalities can identify direct and indirect signs of acute appendicitis, which can vary greatly from patient to patient, particularly in the early stages of the condition.^(3,7)^ The description of these signs in strictly objective terms results in a heterogeneous assessment of the likelihood of acute appendicitis by emergency physicians and surgeons, thereby affecting decision-making regarding further care.^(13)^

To address this challenge, reporting systems have been developed and validated for acute appendicitis on CT scans.^(14–16)^ Ultrasound findings have also been described in acute appendicitis, specifically in children.^(17,18)^ Drawing inspiration from established systems such as the Breast Imaging Reporting and Data System^(19)^ and Thyroid Imaging Reporting and Data System,^(20)^ the Appendix Imaging Reporting and Data System (APENDIC-RADS) is an initiative aiming to fill this gap by standardizing the ultrasonography assessment of acute appendicitis in all age groups.

## OBJECTIVE

To introduce the Appendix Imaging Reporting and Data System (APENDIC-RADS) to standardize the reporting of appendix ultrasound findings.

## METHODS

### Study design and participant selection

In this retrospective study, we included consecutive patients with suspected appendicitis who underwent abdominal ultrasonography between December 1, 2019, and January 31, 2020. This study was conducted in the emergency department of a private general hospital in São Paulo, Brazil.

### Study population

The study targeted individuals of any age who presented with suspected acute appendicitis and underwent abdominal ultrasonography in the emergency department. There were no exclusion criteria in this study.

### Outcome and follow-up

The primary outcome was confirmation of acute appendicitis in surgical cases through histopathological appendix evaluation. In cases where no clinical or surgical treatment was reported in the medical records within 30 days after ultrasonography, the absence of clinical or surgical treatment for acute appendicitis was confirmed through a follow-up phone call conducted by the authors.

### Study procedures

All ultrasonography examinations were conducted as part of routine care by radiologists. The standard protocol involved convex and linear probes (C 1-5 MHz, L 10-12 MHz, L 9-12 MHz) for craniocaudal scans, covering the transition from the transverse and ascending colon to the cecum and the right iliac region. Epiq 7 G (Philips Ultrasound Inc., Bothell, WA), and GE LOGIQ S8 (General Electric Health Care) were the ultrasound devices used. We identified study patients by searching the hospital's electronic storage system using ‘acute appendicitis’ as a keyword in the examination reports.

**Figure 1:**
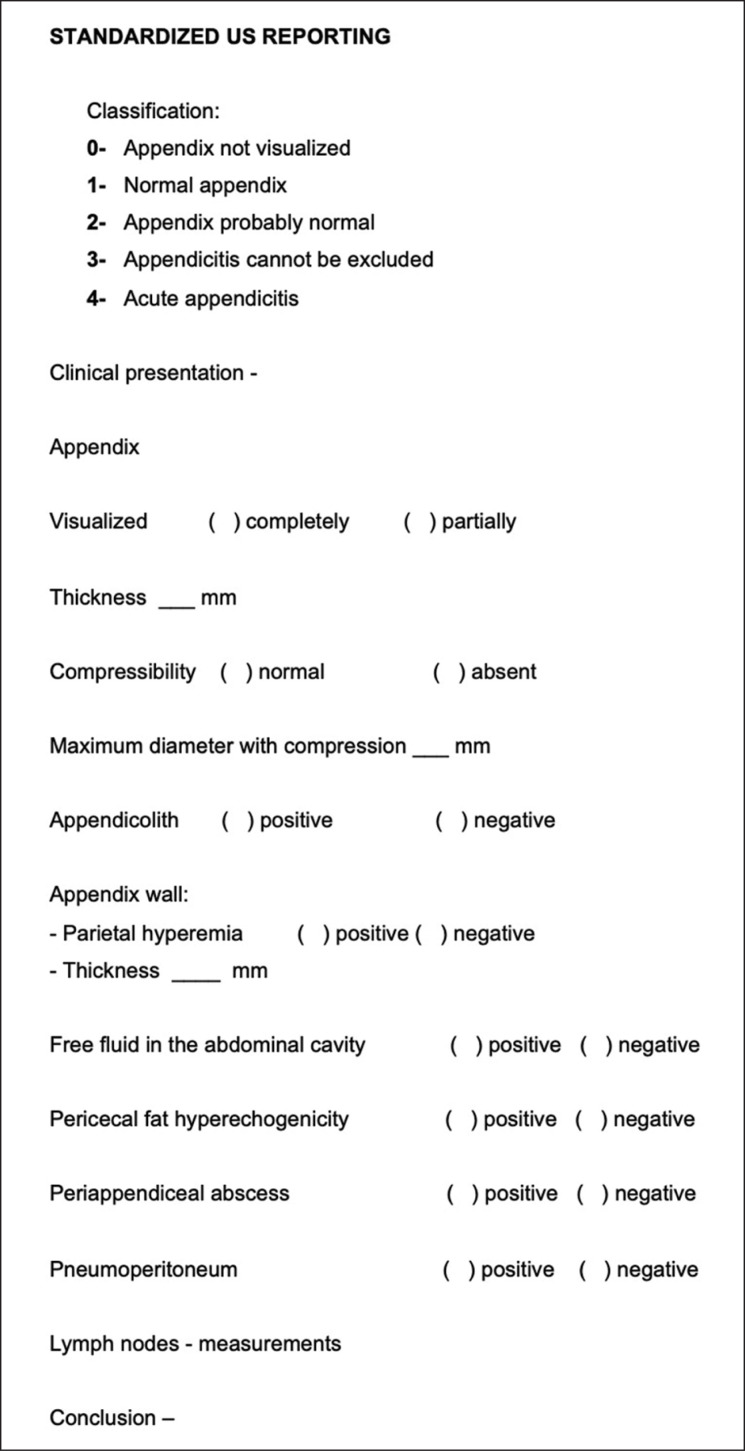
APENDIC-RADS ultrasonography template illustrating categorization from non-visible appendix to confirmed acute appendicitis

The APENDIC-RADS system was developed from a comprehensive literature review^(3–5,7,21–27)^ and clinical expertise. It was incorporated into standard abdominal ultrasonography reports starting December 1, 2019, following a preliminary pilot analysis. The ultrasonography template is available in figure 1. The categorization spans from APENDIC-RADS 0, indicating an appendix that remains not visible despite attempts to locate it; APENDIC-RADS 1, representing a normal appendix; APENDIC-RADS 2, denoting an appendix that is probably normal - partially visualized with normal features; APENDIC-RADS 3, suggesting that appendicitis cannot be excluded due to indeterminate features, making appendicitis inconclusive; and APENDIC-RADS 4, confirming the presence of acute appendicitis. Further imaging details are provided in table 1.

**Table 1:** Radiological findings for each APENDIC-RADS category

Radiological findings	APENDIC-RADS category				
	0	1	2	3	4
Appendix view	None	Complete	Partial	Complete or partial	Complete or partial
Maximum transverse diameter of the appendix under compression larger than 6 mm and loss of compressibility	Not assessable	Absent	Absent	Absent	Present
Direct appendicitis signs – Appendicolith – Wall: thickening larger than 3 mm and/or loss of stratification of parietal layers and/or increased periappendiceal echogenicity and/or increased vascularity on color Doppler – Periappendiceal abscess	Not assessable	Absent	Absent	At least one direct or indirect sign is present (except periapendicular abscess)	May be present
Indirect appendicitis signs – Free abdominal fluid – Hyperechogenicity of the pericecal fat – Pneumoperitoneum, gas outside the intestinal loop – Locoregional adenopathy	Not assessable	Absent	May be present (except pneumoperitoneum)	At least one direct or indirect sign is present (except periapendicular abscess)	May be present

### Data collection

Demographic and clinical information were extracted from patient charts. These data included age, sex, symptoms (fever, nausea, vomiting, anorexia, and diarrhea), presence of sudden decompression in the right iliac fossa (Blumberg sign), age-adjusted leukocytosis, and left shift. Data from the selected episodes were recorded using the Research Electronic Data Capture system.^(28,29)^

### Statistics

Considering that the general population prevalence of acute appendicitis is 8%^(3,28)^ we calculated a sample size of 707 ultrasonography examinations would be required. This size was determined to ensure 95% confidence in correlating ultrasonography signs with acute appendicitis diagnoses, and a maximum sampling error of 2%.

Descriptive statistics were stratified according to acute appendicitis diagnosis. Data are presented as mean and standard deviation or median and interquartile range, depending on their distribution. The analyses included the χ^2^ test, Fisher's exact test, and the likelihood ratio test. Additionally, we constructed a receiver operating characteristic (ROC) curve and a calibration belt. The sensitivity, specificity, positive predictive value, and negative predictive value of the APENDIC-RADS categories for the diagnosis of acute appendicitis were also assessed. The analyses were performed using IBM-SPSS for Windows version 22.0 and STATA software (StataCorp, College Station, TX) version 16.0.

### Ethical considerations

The study was approved by the ethics committee of *Hospital Israelita Albert Einstein* (CAAE: 29649520.2.0000.0071; # 4.327.131). The requirement for informed consent was waived. No funding was received for this study.

## RESULTS

During the study period from December 1, 2019, to January 31, 2020, we evaluated 747 patients who underwent abdominal ultrasonography for suspected acute appendicitis in the emergency department. The median age of participants was 21 years, with a notable predominance of women (58.6%). The clinical presentation analysis revealed that the most prevalent signs and symptoms among those suspected of having acute appendicitis included nausea and/or vomiting (47.7%), followed by diarrhea (29.7%) and pain in the right iliac fossa (26.4%).

Among the participants diagnosed with acute appendicitis, 52% were male. The most frequently reported symptoms in the diagnosed cases were nausea and/or vomiting (60%) and pain in the right iliac fossa (54%). Furthermore, patients with appendicitis showed a significantly higher age-adjusted prevalence of leukocytosis and symptoms such as right iliac fossa pain and sudden decompression in the right iliac fossa (p<0.001), underscoring their diagnostic value. Baseline characteristics of the study population are shown in table 2.

**Table 2:** Baseline characteristics of the study population

Characteristics		Total n=747	Appendicitis ruled out n=697	Confirmed appendicitis n=50	p value
Female sex, n (%)		438 (58.6)	414 (59.4)	24 (48)	0.106
Age - years		21±16	21±17	22±15	0.723
Age group, n (%)					
<2 years		15 (2.0)	15 (2.2)	0 (0)	0.180
2 to 4 years		54 (7.2)	52 (7.5)	2 (4.0)	
	5 to 10 years	181 (24.2)	170 (24.4)	11 (22.0)	
	11 to 16 years	113 (15.1)	104 (14.9)	9 (18.0)	
	17 to 30 years	125 (16.8)	113 (16.2)	12 (24.0)	
	31 to 45 years	184 (24.6)	171 (24.5)	13 (26.0)	
	46 to 60 years	39 (5.2)	36 (5.2)	3 (6.0)	
	>60 years	36 (4.8)	36 (5.2)	0 (0)	
Signs and symptoms, n (%)					
	Fever	184 (24.6)	177 (25.4)	7 (14.0)	0.110
	Nausea and/or vomiting	356 (47.7)	326 (46.8)	30 (60.0)	0.042
	Anorexia	72 (9.6)	65 (9.3)	7 (14.0)	0.155
	Diarrhea	222 (29.7)	212 (30.4)	10 (20.0)	0.181
	Right iliac fossa pain	197 (26.4)	170 (24.4)	27 (54.0)	<0.001
	Sudden decompression	37 (5.0)	25 (3.6)	12 (24.0)	<0.001
Leukocytosis *, n (%)		152 (20.3)	120 (17.2)	32 (64.0)	<0.001

* leukocytosis adjusted for age.

The incidence of acute appendicitis showed significant variation across the APENDIC-RADS categories, with rates of 4.7% in non-visible appendix cases (APENDIC-RADS 0) and 0.7%, 2.2%, 11.5%, and 93.5% for APENDIC-RADS categories 1 to 4, respectively (p<0.001). The distribution of participants according to their APENDIC-RADS classification and the corresponding rates of acute appendicitis are detailed in table 3. The analysis also identified several differential diagnoses for patients without acute appendicitis, including acute gastroenterocolitis, urinary tract infection, mesenteric adenitis, ureterolithiasis, ovarian cysts, and pelvic inflammatory disease, among others.

**Table 3:** Distribution of participants based on the occurrence of acute appendicitis according to the APENDIC-RADS classification

APENDIC-RADS	Total n=747 n (%)	Appendicitis ruled out n=697 n (%)	Confirmed appendicitis n=50 n (%)	p value
0	309 (41.4)	295 (42.3)	14 (28.0)	<0.001
1	292 (39.1)	290 (41.6)	2 (4.0)	
2	89 (11.9)	87 (12.5)	2 (4.0)	
3	26 (3.5)	23 (3.3)	3 (6.0)	
4	31 (4.1)	2 (0.3)	29 (58.0)	

Specifically, among patients classified as APENDIC-RADS 3, 16 (61.5%) underwent a subsequent CT scan, which confirmed appendicitis in three cases. The remaining cases that did not undergo CT were advised to maintain clinical observation, and no diagnosis of appendicitis was confirmed in any of them. In this group, a significant association was found between the presence of positive sudden decompression in the right iliac fossa and confirmed acute appendicitis, with an odds ratio of 16.5 [95% confidence interval (95%CI)=2.1-132.5; p=0.008)]. Conversely, symptoms such as nausea/vomiting, fever, or right iliac fossa pain did not show a significant correlation with appendicitis diagnosis in this category, suggesting their limited predictive value in isolation.

The APENDIC-RADS model demonstrated an area under the ROC curve of 0.950 (95% CI=0.899-1). The model achieved a sensitivity of 87.9% and specificity of 93.8% in excluding acute appendicitis in lower categories (APENDIC-RADS 1 and 2) compared to the confirmed diagnoses in APENDIC-RADS 4. The positive predictive value was 93.5%, and the negative predictive value was 99%. The model calibration was assessed using a calibration belt. The results indicate that the observed proportions aligned well with the 95%CIs of the predicted probabilities, suggesting a good match between the expected and observed outcomes.

## DISCUSSION

The challenge of accurately diagnosing acute appendicitis in emergency care settings remains significant, with imaging playing a pivotal role.^(2,9)^ Our study reinforces the value of ultrasonography, not only for its safety advantage by avoiding radiation exposure but also for its good diagnostic accuracy.^(10–12)^ With an appendix visualization rate of 58.5%, our results are consistent with those in the existing literature, affirming the proficiency of our radiology team in employing high-standard ultrasonography imaging.^(30)^

The introduction of the APENDIC-RADS system marks a significant advancement in the standardization of ultrasonography reporting for suspected acute appendicitis across all age groups. Demonstrating remarkable diagnostic accuracy (AUROC of 0.950), our findings suggest that the APENDIC-RADS is a robust tool that enhances diagnostic confidence. The system's high sensitivity, specificity, and predictive values facilitate accurate decision-making, from confidently excluding appendicitis in patients in the APENDIC-RADS 1 category to identifying patients who may benefit from further clinical observation or advanced imaging through the APENDIC-RADS 2 and 3 categories and confirming the diagnosis in the APENDIC-RADS 4 category. Although the APENDIC-RADS 2 category did not indicate a significantly higher likelihood of appendicitis than APENDIC-RADS 1 in our sample, its importance cannot be understated. This category allows for careful observation or progression on CT scans. In a previous study, the reported rate of acute appendicitis in partially visualized appendices was 15.6%, indicating that it is a distinct category when compared to fully visualized appendices.^(31)^ Given the diverse anatomical variations in the location of the appendix tip, such as the retrocecal, pelvic, and pre- or post-ileal positions, it is crucial to evaluate the entire length of the appendix to exclude the possibility of initial-stage appendicitis.^(32,33)^ Moreover, the APENDIC-RADS 3 category, which primarily consists of indeterminate features, has proven to be a reliable tool for prompting further investigation in cases where physical examination suggests appendicitis. We recommend re-evaluating the patient in a few hours when clinical suspicion is not high. This reassurance about its accuracy should instill confidence in its use.

Given that out of 747 patients, only 100 underwent CT, this underscores the importance of the APENDIC-RADS classification in avoiding radiation exposure. It is particularly valuable in the emergency setting, especially for pediatric patients.

In addition to its diagnostic utility, the APENDIC-RADS system enhances communication among healthcare teams, patients, and their families. Furthermore, it aligns with the contemporary demands of the new social context of medicine, in which reports are no longer just between doctors and patients but involve media channels, films, and image digitization.^(34)^

Previously, two pediatric-focused ultrasound reporting scores have been developed.^(17,18)^ Table 4 provides a comparison of these scores with the APENDIC-RADS. Unlike the Appy score, our methodology streamlines the classification by not distinguishing between confirmed cases based on perforation status.^(17)^ Furthermore, we reorganized the categories into a sequence that more intuitively indicates the likelihood of appendicitis, except for category 0, which denotes a non-visible appendix. Unlike Sola et al.'s method, our system introduces a specific category for partially visualized appendices.^(18)^ These modifications were designed to align with our understanding of priorities and have been validated across all age groups.

**Table 4:** Comparative Analysis of Pediatric-Focused Ultrasound Reporting Scores and APENDIC-RADS^(16-18)^

Reporting system	Appy-Score	Sola et al.^(18)^	APENDIC-RADS
Description	1. Completely visualized normal-appearing appendix with no ancillary findings to suggest appendicitis 2. Partially visualized normal-appearing appendix with no ancillary findings to suggest appendicitis 3. Non-visualized appendix with no ancillary findings to suggest appendicitis 4. Equivocal 5a. Non-perforated acute appendicitis 5b. Perforated appendicitis	Category 1. Negative for appendicitis Category 2. Appendix not visualized without any secondary signs of appendicitis Category 3. Appendix not visualized with secondary signs of appendicitis Category 4. Findings consistent with appendicitis	0. Appendix not visualized 1. Normal appendix 2. Appendix probably normal: partially visualized with normal features 3. Appendicitis cannot be excluded: indeterminate features, making appendicitis inconclusive 4. Acute appendicitis

**Figure 2:**
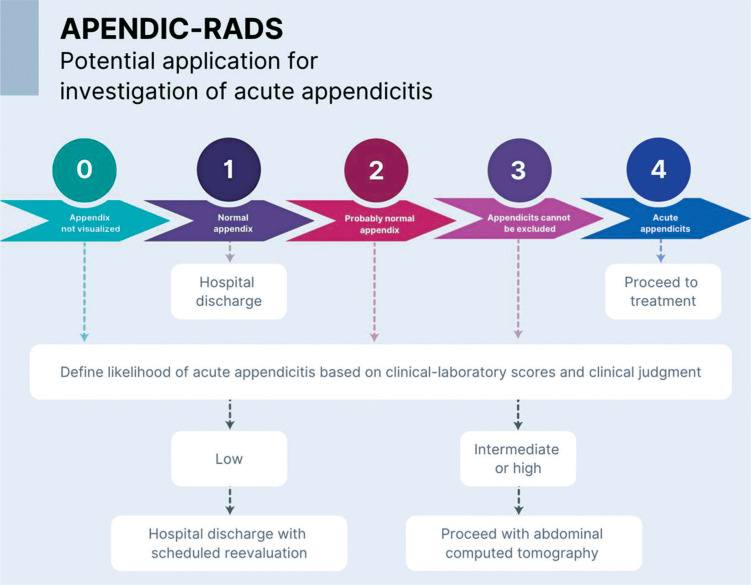
Potential application of APENDIC-RADS for investigation of acute appendicitis

Prospects for APENDIC-RADS include its integration with clinical assessment tools, which, although not evaluated in our study, represent an important facet of the appendicitis diagnosis. Clinical scores should be used to evaluate the need for imaging, according to the guidelines of the World Society of Emergency Surgery.^(12)^ The most widely used tools in clinical practice are the Alvarado, Raja Isteri Pengiran Anak Saleha Appendicitis, Adult Appendicitis Score (AAS), Appendicitis Inflammatory Response (AIR), and Pediatric Appendicitis Score.^(12,35–38)^ AIR and AAS have the best performance scores.^(12)^ Figure 2 illustrates the proposed future integration of the APENDIC-RADS with clinical tools, which requires further evaluation.

Our study also highlights the potential for enhancing diagnostic proficiency through point-of-care ultrasound (POCUS) training for emergency physicians. A meta-analysis of studies that utilized POCUS for diagnosing acute appendicitis revealed that emergency physicians generally achieve a sensitivity of 80% and specificity of 90%. However, the findings of these studies showed considerable variability.^(39)^ Notably, a study by Fox et al.^(40)^ highlighted a significant disparity: emergency physicians skilled in general ultrasonography use but lacking specific training for appendiceal evaluation only attained a sensitivity of 39% for diagnosing acute appendicitis, accompanied by a high rate of 27.1% false-negative exams. Similar to our study's methodology, these studies predominantly used the presence of a non-compressible appendix exceeding 6 mm in diameter as the primary diagnostic criterion.^(37,38)^ Incorporating comprehensive ultrasonographic indicators into routine evaluations can improve outcomes and facilitate broader team training.

However, it is important to acknowledge the limitations of this study. The retrospective design and evaluator-dependent nature of ultrasonographic examinations introduce potential biases. Additionally, the absence of external validation for the APENDIC-RADS calls for a cautious interpretation of its generalizability. Future studies focusing on external validation and the association of APENDIC-RADS with clinical-laboratory models for the prediction of acute appendicitis are necessary to establish a validated diagnostic algorithm and the generalization of their application.

## CONCLUSION

APENDIC-RADS categorization demonstrated excellent performance in standardizing the ultrasonography-determined probability of acute appendicitis. This suggests its potential to enhance diagnostic accuracy, facilitate better communication among physicians, increase transparency, and standardize patient management. Future studies should explore its integration into clinical practice and assess its impact on clinical outcomes.

## References

[1] Ferris M, Quan S, Kaplan BS, Molodecky N, Ball CG, Chernoff GW (2017). The Global Incidence of Appendicitis: a Systematic Review of Population-based Studies. Ann Surg.

[2] Moris D, Paulson EK, Pappas TN (2021). Diagnosis and Management of Acute Appendicitis in Adults: A Review. JAMA.

[3] Botter L, Oliveira GR, Farias JL, Maurano A, Garcia RG, Queiroz MR (2005). Ultrasonography in the diagnosis of acute appendicitis. einstein (Sao Paulo).

[4] Wiersma F, Srámek A, Holscher HC (2005). US features of the normal appendix and surrounding area in children. Radiology.

[5] Lembcke B (2016). Ultrasonography for acute appendicitis - the way it looks today. Z Gastroenterol.

[6] Yang Z, Sun F, Ai S, Wang J, Guan W, Liu S (2019). Meta-analysis of studies comparing conservative treatment with antibiotics and appendectomy for acute appendicitis in the adult. BMC Surg.

[7] Rettenbacher T, Hollerweger A, Macheiner P, Gritzmann N, Daniaux M, Schwamberger K (2003). Ovoid shape of the vermiform appendix: a criterion to exclude acute appendicitis-evaluation with US. Radiology.

[8] Hwang ME (2018). Sonography and Computed Tomography in Diagnosing Acute Appendicitis. Radiol Technol.

[9] Arruzza E, Milanese S, Li LS, Dizon J (2022). Diagnostic accuracy of computed tomography and ultrasound for the diagnosis of acute appendicitis: a systematic review and meta-analysis. Radiography (Lond).

[10] Auxier JA, Dickson HW (1983). Guest editorial: concern over recent use of the ALARA philosophy. Health Phys.

[11] Oestreich AE (2014). RSNA centennial article: ALARA 1912: "As low a dose as possible" a century ago. Radiographics.

[12] Di Saverio S, Podda M, De Simone B, Ceresoli M, Augustin G, Gori A (2020). Diagnosis and treatment of acute appendicitis: 2020 update of the WSES Jerusalem guidelines. World J Emerg Surg.

[13] Godwin BD, Simianu VV, Drake FT, Dighe M, Flum D, Bhargava P (2015). Is there a need to standardize reporting terminology in appendicitis?. Ultrasound Q.

[14] Kim HC, Yang DM, Kim SW, Park SJ (2012). Reassessment of CT images to improve diagnostic accuracy in patients with suspected acute appendicitis and an equivocal preoperative CT interpretation. Eur Radiol.

[15] Godwin BD, Drake FT, Simianu VV, Shriki JE, Hippe DS, Dighe M (2015). A novel reporting system to improve accuracy in appendicitis imaging. AJR Am J Roentgenol.

[16] Simianu VV, Shamitoff A, Hippe DS, Godwin BD, Shriki JE, Drake FT (2017). The Reliability of a Standardized Reporting System for the Diagnosis of Appendicitis. Curr Probl Diagn Radiol.

[17] Fallon SC, Orth RC, Guillerman RP, Munden MM, Zhang W, Elder SC (2015). Development and validation of an ultrasound scoring system for children with suspected acute appendicitis. Pediatr Radiol.

[18] Sola R, Theut SB, Sinclair KA, Rivard DC, Johnson KM, Zhu H (2018). Standardized reporting of appendicitis-related findings improves reliability of ultrasound in diagnosing appendicitis in children. J Pediatr Surg.

[19] American College of Radiology (2013). Breast Imaging Reporting and Data System (BI-RADS).

[20] Horvath E, Majlis S, Rossi R, Franco C, Niedmann JP, Castro A (2009). An ultrasonogram reporting system for thyroid nodules stratifying cancer risk for clinical management. J Clin Endocrinol Metab.

[21] Park NH, Park CS, Lee EJ, Kim MS, Ryu JA, Bae JM (2007). Ultrasonographic findings identifying the faecal-impacted appendix: differential findings with acute appendicitis. Br J Radiol.

[22] Je BK, Kim SB, Lee SH, Lee KY, Cha SH (2009). Diagnostic value of maximal-outer-diameter and maximal-mural-thickness in use of ultrasound for acute appendicitis in children. World J Gastroenterol.

[23] Trout AT, Sanchez R, Ladino-Torres MF (2012). Reevaluating the sonographic criteria for acute appendicitis in children: a review of the literature and a retrospective analysis of 246 cases. Acad Radiol.

[24] Searle AR, Ismail KA, Macgregor D, Hutson JM, Hutsona MJ (2013). Changes in the length and diameter of the normal appendix throughout childhood. J Pediatr Surg.

[25] Coyne SM, Zhang B, Trout AT (2014). Does appendiceal diameter change with age? A sonographic study. AJR Am J Roentgenol.

[26] Prendergast PM, Poonai N, Lynch T, McKillop S, Lim R (2014). Acute appendicitis: investigating an optimal outer appendiceal diameter cut-point in a pediatric population. J Emerg Med.

[27] Wagner M, Tubre DJ, Asensio JA (2018). Evolution and Current Trends in the Management of Acute Appendicitis. Surg Clin North Am.

[28] Harris PA, Taylor R, Thielke R, Payne J, Gonzalez N, Conde JG (2009). Research electronic data capture (REDCap)-a metadata-driven methodology and workflow process for providing translational research informatics support. J Biomed Inform.

[29] Harris PA, Taylor R, Minor BL, Elliott V, Fernandez M, O’Neal L, McLeod L, Delacqua G, Delacqua F, Kirby J, Duda SN, REDCap Consortium (2019). The REDCap consortium: Building an international community of software platform partners. J Biomed Inform.

[30] Chang ST, Jeffrey RB, Olcott EW (2014). Three-step sequential positioning algorithm during sonographic evaluation for appendicitis increases appendiceal visualization rate and reduces CT use. AJR Am J Roentgenol.

[31] Ross MJ, Liu H, Netherton SJ, Eccles R, Chen PW, Boag G (2014). Outcomes of children with suspected appendicitis and incompletely visualized appendix on ultrasound. Acad Emerg Med.

[32] Butler P, Mitchell A, Healy JC (2012). Applied Radiological Anatomy.

[33] Rosse C, Gaddum-Rosse P, Hollinshead WH (1997). Hollinshead's textbook of anatomy.

[34] Cappola AR, Cohen KS (2024). Strategies to Improve Medical Communication. JAMA.

[35] Alvarado A (1986). A practical score for the early diagnosis of acute appendicitis. Ann Emerg Med.

[36] Chong CF, Adi MI, Thien A, Suyoi A, Mackie AJ, Tin AS (2010). Development of the RIPASA score: a new appendicitis scoring system for the diagnosis of acute appendicitis. Singapore Med J.

[37] Sammalkorpi HE, Mentula P, Leppäniemi A (2014). A new adult appendicitis score improves diagnostic accuracy of acute appendicitis-a prospective study. BMC Gastroenterol.

[38] Andersson M, Kolodziej B, Andersson RE, STRAPPSCORE Study Group (2017). Randomized clinical trial of Appendicitis Inflammatory Response score-based management of patients with suspected appendicitis. Br J Surg.

[39] Matthew Fields J, Davis J, Alsup C, Bates A, Au A, Adhikari S (2017). Accuracy of Point-of-care Ultrasonography for Diagnosing Acute Appendicitis: a Systematic Review and Meta-analysis. Acad Emerg Med.

[40] Fox JC, Hunt MJ, Zlidenny AM, Oshita MH, Barajas G, Langdorf MI (2007). Retrospective analysis of emergency department ultrasound for acute appendicitis. Cal J Emerg Med.

